# Ending the doctor–patient relationship

**DOI:** 10.1177/10398562231159544

**Published:** 2023-03-03

**Authors:** Kirk Lehman, Vinuri Edirisinghe

**Affiliations:** 3555 Bond University, Robina, QLD, Australia; 3555 Bond University, Robina, QLD, Australia

**Keywords:** therapeutic relationship, communication, termination, duty of care, documentation

## Abstract

**Objective:**

Terminating a therapeutic relationship can be particularly challenging and onerous for the treating medical practitioner. There are multiple reasons why a practitioner may desire to terminate the relationship, ranging from inappropriate behaviour and assault through to threatened or actual litigation. This paper provides psychiatrists as well as all doctors and support staff who work alongside them with a simple, step-by-step visual guide to terminating a therapeutic relationship, giving due consideration to their professional and legal obligations in line with common recommendations by medical indemnity organisations.

**Conclusions:**

If a practitioner’s ability to manage the patient is inadequate or compromised due to an emotional, financial, or legal circumstance, it is reasonable to consider termination of the relationship. Practical steps such as taking contemporaneous notes, writing to the patient and their primary care doctor, ensuring continuity of healthcare, and communicating with authorities where appropriate have been identified as components commonly recommended by medical indemnity insurance organisations.

Terminating a therapeutic relationship is onerous and can unfortunately at times feel solely one sided with the burden lying squarely on the shoulders of the treating medical practitioner. While patients have the discretion to leave their treating medical practitioner at will, a medical practitioner’s decision to potentially end a therapeutic relationship is burdened by a number of considerations so as to uphold their duty of care.

## Why would a doctor wish to cease seeing a patient and terminate their care?

The Medical Board of Australia stipulates that when the doctor–patient relationship becomes ineffective or otherwise compromised, it may be appropriate to terminate the relationship.^1^

Ideally the relationship ceases by mutual agreement for reasons such as the patient relocating to a new area or no longer requiring treatment. Alternatively, the patient may be directed to another medical practitioner who will assume care via referral.^2^ Such circumstances are typically not problematic but should still be well documented.

On occasion however, the reasons for considering termination are far more challenging and confronting, involving the irrevocable breakdown in the therapeutic relationship.

These may include, but are not limited, to the following circumstances:

1. The patient exhibiting inappropriate or unacceptable behaviour^3^ that violates professional boundaries including verbal abuse, sexual/sexist comments, or advances.
2. The patient committing criminal acts, including falsifying documentation.
3. The doctor’s recognition that for whatever reason, they are unable to provide care in the patient’s best interests.
4. The patient exhibiting coercive drug-seeking behaviour.
5. The patient attempting to coerce the medical practitioner into providing a treatment contrary to the medical practitioner’s professional opinion.
6. The patient attempting to coerce the treating practitioner into falsifying documentation, including but not limited to backdating medical certificates or drafting materially false medical reports.
7. The patient’s failure to pay fee accounts.
8. The patient has made an AHPRA complaint or is pursuing litigation against the medical practitioner, thereby potentially compromising the medical practitioner’s ability to provide impartial care.

Certain circumstances may warrant the immediate termination of the therapeutic relationship, including if the patient has threatened or assaulted the medical practitioner.^4^

More commonly however, practitioners are faced with circumstances where the decision to terminate is not obvious nor simple. In these situations, practitioners are often able to foresee how the therapeutic relationship can deteriorate beyond repair, particularly without course correction.

Common examples of such circumstances may include, but are not limited, to the following:

1. Situations involving transference or countertransference.
2. A real, or perceived lack of authenticity or effort by the patient to address the issues of concern, or to attend scheduled appointments.
3. The patient being difficult/demanding and/or is continuously non-compliant with management recommendations.
4. Incompatibility between the patient’s expectations and the doctor’s consultation style, approach and treatment plan(s).

In such cases, discussions with colleagues and/or a medical indemnity provider is recommended to source alternative strategies to salvage a deteriorating therapeutic relationship.

In summary, if the medical practitioner’s ability to manage the patient is inadequate or compromised due to an emotional, financial, or legal circumstance, it is reasonable to consider termination of the therapeutic relationship and to facilitate the referral of the patient to a medical practitioner better suited to provide the patient with the care required. Termination of the doctor–patient relationship on medically irrelevant grounds such as those including the patient’s race, religion, sex, gender, or disability would be considered unlawful discrimination in accordance with anti-discrimination legislation.^5,6^ It should also be noted that emergency care should never be withheld from a patient.

## How to end a therapeutic relationship

**Figure 1.**
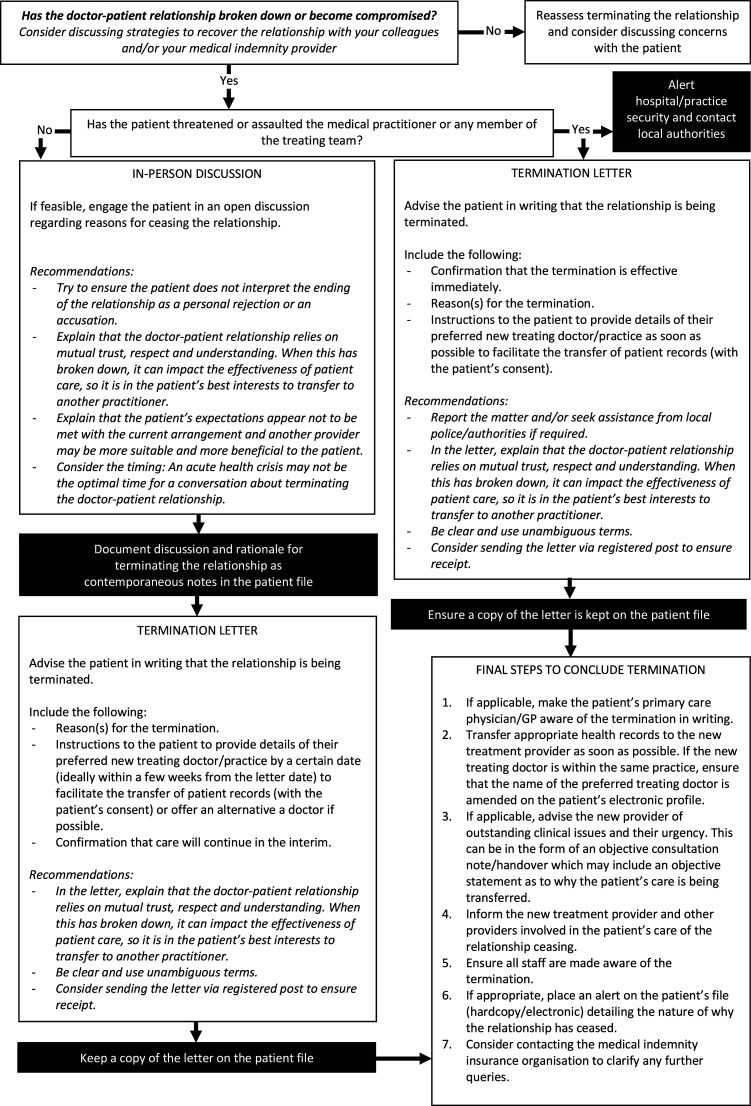
How to end a therapeutic relationship.

The Medical Board of Australia identifies that good medical practice involves ensuring that the patient is adequately informed of the clinician’s decision and arrangements be facilitated to ensure continuity of care, including passing on relevant clinical information.^7^ This view appears to have guided medical indemnity insurance organisations to identify a number of practical components that must be completed in order to successfully terminate the therapeutic relationship without breaching professional and legal obligations. The flow chart in Figure 1 is a consolidation of components commonly agreed upon by the medical indemnity insurance organisations in Australia.^3,8–11^ All correspondence and discussions must be documented in the patient’s file. Consultation with one’s medical indemnity insurer should always be considered.

## The consequences of poor practice

Poor or ambiguous communication coupled with a failure to set clear boundaries to end the relationship cleanly can result in the patient making a complaint alleging an unreasonable refusal to treat.^12^ If the patient suffers harm due to a failure to provide care, particularly in an emergency or prior to care being arranged elsewhere, the original treating doctor may be held liable.^13^ Failure to notify the patient’s primary care physician/GP (if applicable) of the termination may also lead to poor health outcomes. Any changes to the patient’s treating team, including new treating psychiatrists and/or other specialists, must be communicated to the patient’s primary care doctor to ensure continuity of care.
